# Cell specificity dictates similarities in gene expression in multiple sclerosis, Parkinson’s disease, and Alzheimer’s disease

**DOI:** 10.1371/journal.pone.0181349

**Published:** 2017-07-17

**Authors:** Yuichiro Itoh, Rhonda R. Voskuhl

**Affiliations:** Department of Neurology, David Geffen School of Medicine, University of California, Los Angeles, California, United States of America; Instituto Cajal-CSIC, SPAIN

## Abstract

Drug repurposing is an efficient approach in new treatment development since it leverages previous work from one disease to another. While multiple sclerosis (MS), Parkinson’s disease (PD), and Alzheimer’s disease (AD) are all neurodegenerative diseases of the central nervous system (CNS) and differ in many clinical and pathological aspects, it is possible that they may share some mechanistic features. We hypothesized that focusing on gene expression in a CNS cell type specific manner might uncover similarities between diseases that could be missed using whole tissue gene expression analyses. We found similarities and differences in gene expression in these three distinct diseases, depending upon cell type. Microglia genes were increased in all three diseases, and gene expression levels were correlated strongly among these three neurodegenerative diseases. In astrocytes and endothelia, upregulation and correlations were observed only between MS and PD, but not AD. Neuronal genes were down-regulated in all three diseases, but correlations of changes of individual genes between diseases were not strong. Oligodendrocyte showed gene expression changes that were not shared among the three diseases. Together these data suggest that treatments targeting microglia are most amenable to drug repurposing in MS, PD, and AD, while treatments targeting other CNS cells must be tailored to each disease.

## Introduction

Multiple sclerosis (MS) is an autoimmune disease of the central nervous system (CNS) characterized by demyelination and neurodegeneration. The mechanism triggering MS is not known, although genetic, immunological and environmental factors have been proposed [1, 2]. In the peripheral blood of MS patients, lymphocytes are activated and infiltrate the CNS, causing focal demyelination and axonal damage in white matter, with neuronal and synaptic loss in gray matter. Astrocytes and microglia are activated in both white and gray matter. Disabilities vary greatly between MS patients, and may involve walking, vision, and cognition, to name a few. Cognitive disability is affected in over 60% of MS patients at some point in their disease, is not dependent upon white matter lesion location, and correlates with hippocampal and cerebrocortical gray matter pathology [3, 4]. On the other hand, Alzheimer’s disease (AD) is a neurodegenerative disease that targets gray matter, not white matter. Hippocampus and cerebral cortex demonstrate accumulation of extracellular Aβ (β-amyloid) plaques and intra-neuronal neurofibrillary tau lesions [5]. Aβ is produced by the cleavage of Amyloid β Precursor Protein (APP), and the existence of abundant Aβ plaques is a specific feature of AD. Clinically, AD affects primarily short term memory and slows cognitive processing [6], with partial overlap in cognitive domains affected by MS which primarily affects processing speed but also visual-spatial and verbal memory. Finally, the age of onset is also different between MS and AD, with MS onset generally in the second to third decades of life, and AD in sixth to seventh decades. Parkinson’s disease (PD) is also a major neurodegenerative disease and is defined clinically by the symptoms of akinesia, rigidity, and tremor. In this disease, the loss of dopaminergic neurons in the substantia nigra pars compacta is a major neuropathological feature [7, 8]. In addition, significant brain atrophy occurs in the middle and superior frontal gyrus [9]. The presence of cytosolic Lewy bodies characterized by aggregated α-synuclein is the neuropathological hallmark of PD. Although both environmental and genetic factors contribute to PD, age is the greatest risk factor, similar to AD, but distinct from MS. Cognition is also frequently impaired in PD [10–12].

Due to the technological progress and the establishment of genome databases over the last decade, global analyses of gene expression levels are possible. Such global analyses of gene expression patterns can reveal molecular features and relationships among thousands of genes. In accordance with the increasing number of genome wide gene expression analyses, the GEO database has been populated by numerous microarray and high throughput sequencing data. This permits bioinformatics analyses of published data focusing on novel aspects as well as meta-analysis of multiple studies.

In this study, by analyzing data from MS, PD, and AD, we determine similarities and differences in gene expression of specific CNS cell types in these three diseases and identify the CNS cell type possessing the most similarity among these diseases.

## Materials and methods

### Microarray data

Human superior frontal gyrus microarray data for Multiple sclerosis, Parkinson’s disease, and Alzheimer’s disease were obtained from the GEO database. To our knowledge, superior frontal gyrus data was the only available human CNS data common to all of these three conditions. Multiple sclerosis data (GSE26927: MS, average = 49.4 years of age with range of 42–54 years, disease duration of 16–33 years; NL, average = 53.1 years of age with range of 46–59 years) [13, 14]: MS = 5 males and 5 females, and age matched normal controls = 6 males and 4 females. Parkinson’s disease (GSE8397: PD, average = 78.2 years of age with range of 68–87 years; NL, average = 72.0 years of age with range of 57–81 years) [15]: PD = 5 and age matched normal control = 3. In Alzheimer’s disease data (GSE48350) [16], we eliminated the samples under 70 years of age from the normal control group, and focused on normals at 71–99 years of age to match the Alzheimer’s disease patients age. This filtration step reduced the number of samples (AD average = 87.1 years of age with range of 74–95 years: NL, average = 83.4 years of age with range of 70–99 years): AD = 7 males and 14 females, and age matched normal controls = 9 males and 13 females.

### Data analysis

Statistical analyses and production of figures were performed in R (R Core Team, 2016, http://www.R-project.org/). The microarray data sets from GEO database were quantile normalized before running any analysis.

The microarray data sets for MS, PD, and AD, as well as age matched controls for each, were from whole tissues which included mixtures of various different cell types. This heterogeneity of brain cells possibly masks the transcriptome signature of disease in each cell type. To create a gene list for each cell type, lists of top 500 enriched genes in 6 CNS cell types (neuron, microglia, astrocyte, endothelia, newly formed oligodendrocyte, and myelinating oligodendrocyte) from the RNA-sequencing transcriptome database [17] were used as a reference gene list. Although this approach does not completely remove the possible contamination of expression levels from the other cell types, the influence of this impurity can be minimized when the analysis is done for a population of genes, not at a single gene level. For the genes which possess multiple probes, the expression levels were averaged, thus each dot in scatter plots represents one single gene. For the box plot, one sample t-test was performed to show the significant difference from log_2_ disease:normal ratios, with 0.0 indicating no gene expression change by disease. In the correlation coefficient analysis, the existence of outliers could influence the result. Thus, to minimize the effect of outliers, winsorization was performed using R package “robustHD” for the calculation of correlation coefficient and its p-value.

## Results

### Cell type specific gene expression changes in MS, PD, and AD

**Fig 1:**
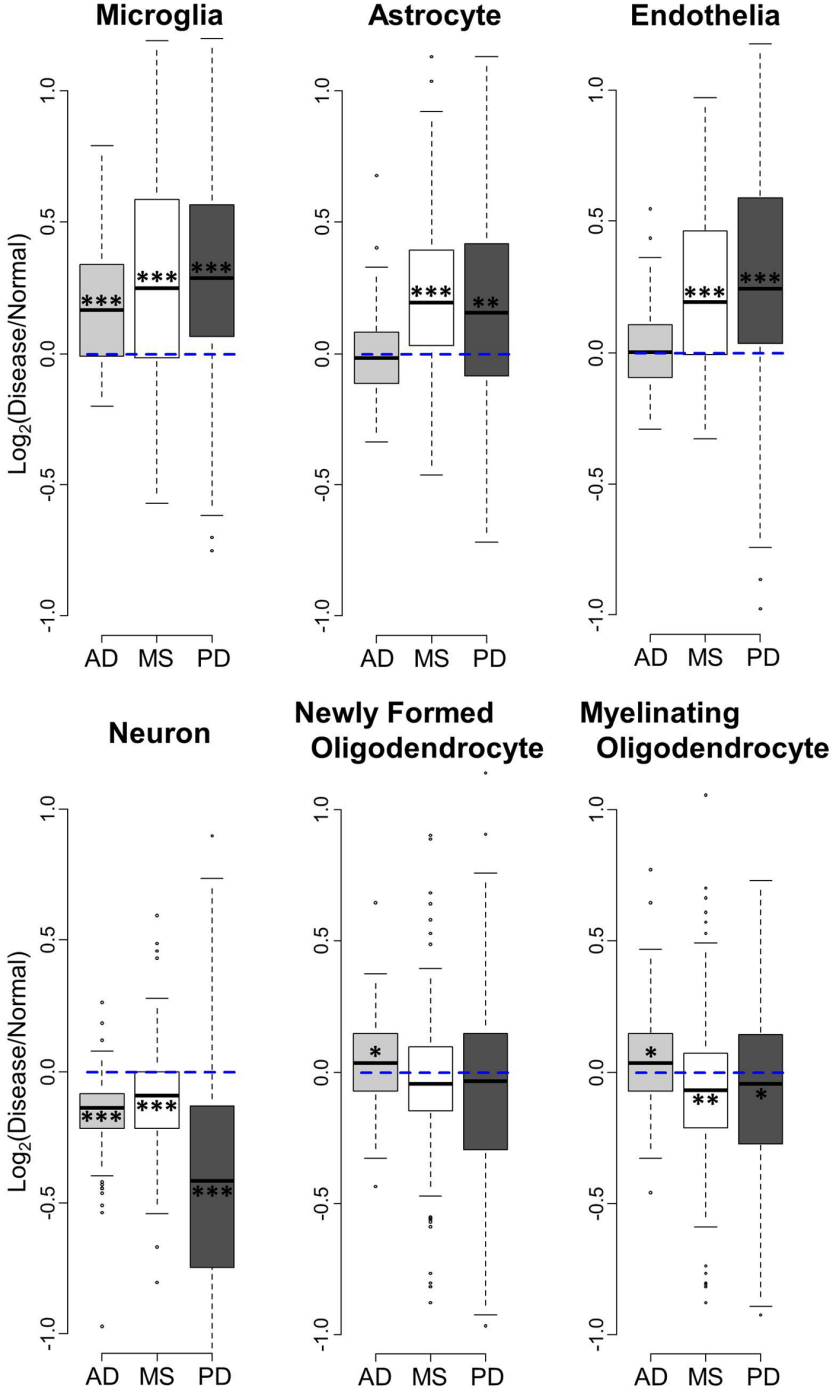
Comparison of disease-related gene expression changes in brains of MS, PD, and AD. Box plots for the log_2_ ratios of gene expression levels between patients and their respective age matched normal controls were made in six different CNS cell types. Blue dashed line shows no gene expression difference between disease and normal. When a gene is above 0 (blue line), the gene expression is upregulated in disease. When it is below 0, the genes are down-regulated. Bold line represents median, and box indicates the 1^st^ and 3^rd^ quartiles. Asterisks: ** p<0.01, **p<0.001, *** p<0.0001 (one sample t-test to show the significant difference from log_2_ ratios = 0).

The microarray data sets analyzed in this study were from whole tissue samples of superior frontal gyrus consisting of heterogeneous cell types. However, by using known cell type enriched gene lists [17], we were able to investigate gene expression of specific CNS cell types in MS, PD, and AD. Using the top 500 enriched gene lists in 6 CNS cell types (neuron, microglia, astrocyte, endothelia, newly formed oligodendrocyte, and myelinating oligodendrocyte), we first examined the overall gene expression change in disease among the three diseases (Fig 1). The gene expression values were normalized by their respective age matched healthy controls by using Disease/Normal ratios to remove the confound of age when comparing between diseases (S1 Table).

Microglia genes were significantly upregulated in all three diseases, while astrocyte and endothelia genes showed upregulation only in MS and PD, but not in AD (Fig 1). Neuronal genes were down-regulated in all three diseases. On the other hand, newly formed and myelinating oligodendrocyte genes did not show shared changes in expression across diseases. Interestingly, MS showed a decrease in myelinating oligodendrocytes, but no decrease in newly formed oligodendrocytes.

### CNS cell type dependent similarities and differences among MS, PD, and AD

**Fig 2:**
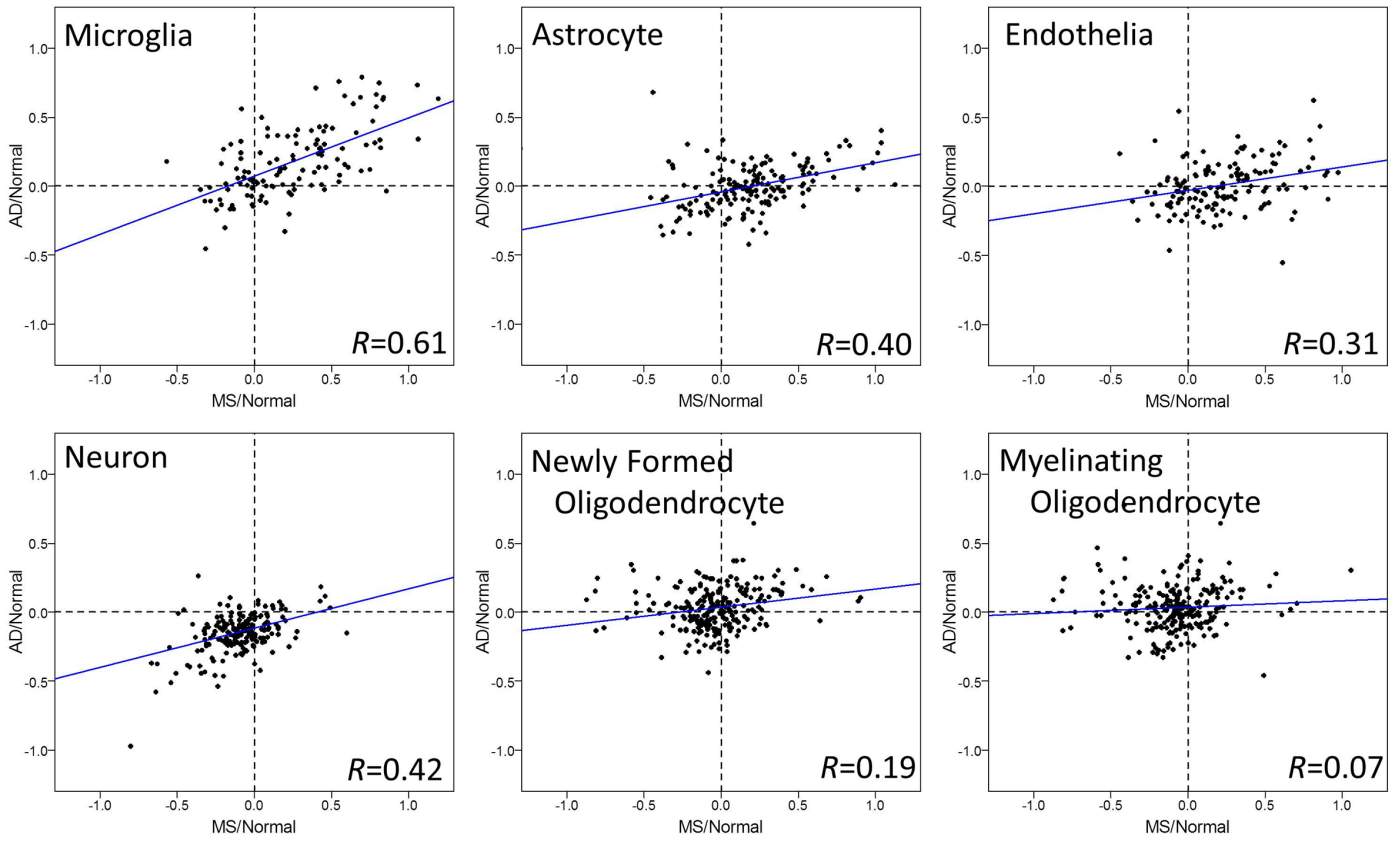
Cell type specific gene expression changes in brains of MS and AD. Log_2_ ratios of gene expression levels between MS or AD patients, normalized for their respective age matched normal controls, are plotted in each CNS cell type. Each dot represents one gene in microarrays. Microglia showed significant positive correlations between MS and AD (p = 5.55e-14), and the strength of relationship was quite strong (correlation = 0.61). Moderate correlations were observed in neurons, astrocytes, and endothelial cells. Regression lines are shown as blue lines.

**Fig 3:**
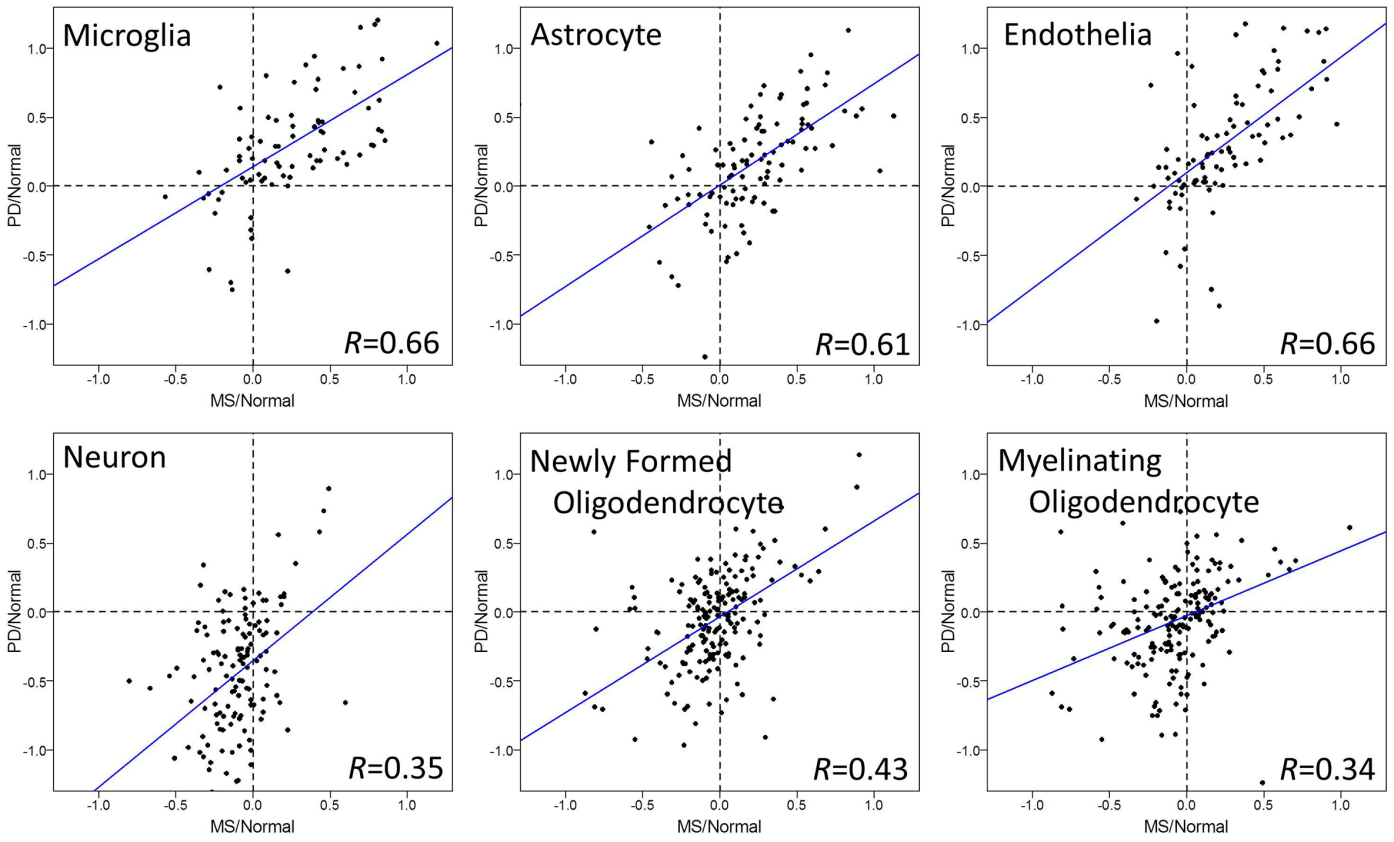
Cell type specific gene expression changes in brains of MS and PD. Log_2_ ratios of gene expression levels between MS or PD patients, normalized for their respective age matched normal controls, are plotted in each CNS cell type. Each dot represents one gene in microarrays. Microglia, astrocytes, and endothelial cells showed significant positive correlations between MS and PD (p<6.21e-12), and the strength of relationship was quite strong (correlation > 0.61). In addition, moderate correlations were observed in the other cell types (neuron, newly formed and myelinating oligodendrocyte). Regression lines are shown as blue lines.

**Fig 4:**
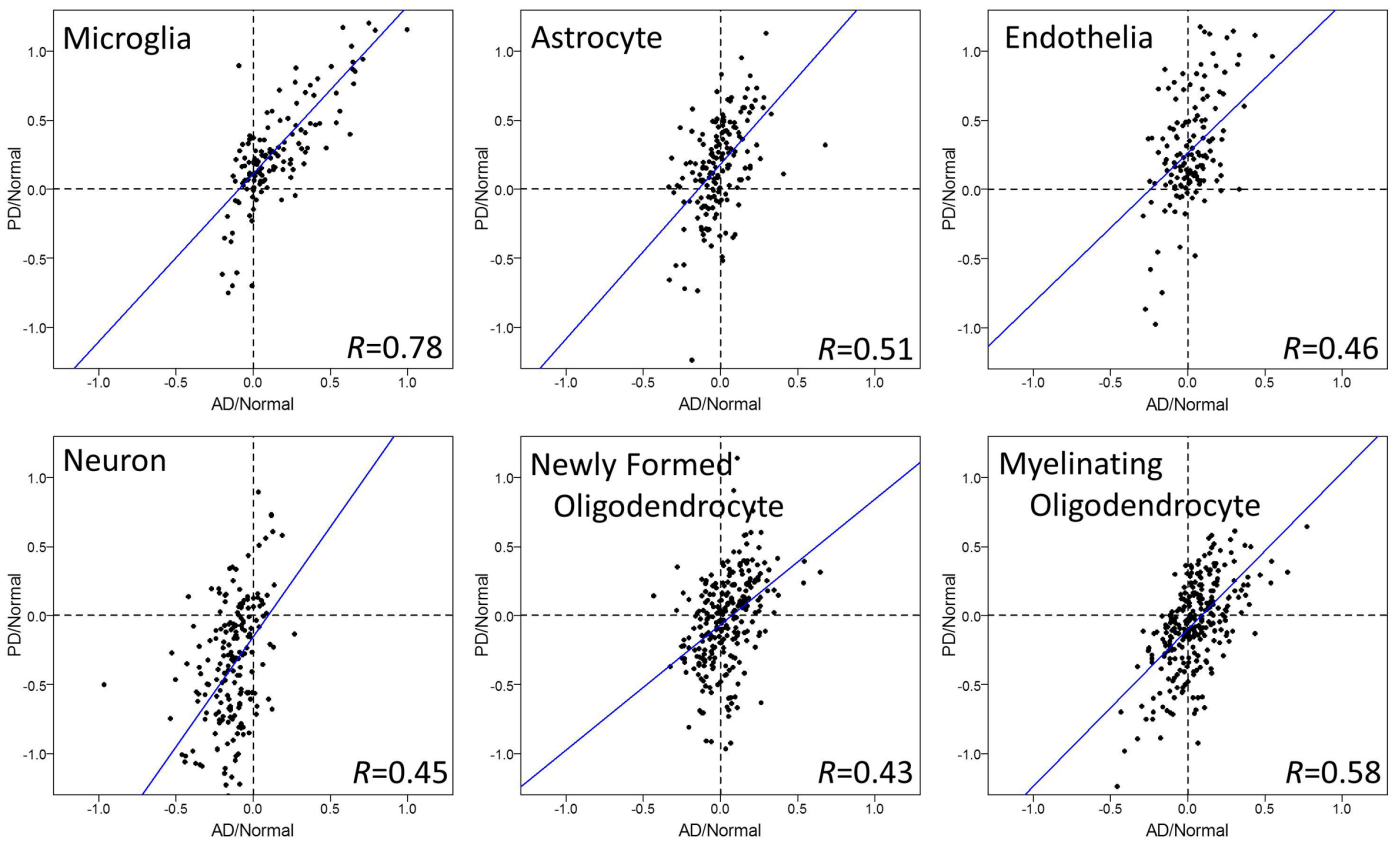
Cell type specific gene expression changes in brains of AD and PD. Log_2_ ratios of gene expression levels between AD or PD patients, normalized for their respective age matched normal controls, are plotted in each CNS cell type. Each dot represents one gene in microarrays. Microglia showed significant positive correlations between AD and PD (p = 0), and the strength of relationship was very strong (correlation = 0.78). Moderate correlations were also observed in the other cell types, however, the gene expression changes in AD were not as strong as the ones in PD. Regression lines are shown as blue lines.

To evaluate the relationship among the diseases further, we tested correlations of cell type specific gene expression between diseases, each normalized to their respective age matched healthy controls (Figs 2, 3 and 4).

Microglia genes showed a significant and strong positive correlation of gene expression change by disease in all three comparisons: MS and AD (Fig 2), MS and PD (Fig 3), AD and PD (Fig 4). Most of the genes were upregulated in these three diseases, consistent with data in Fig 1. The degrees of upregulation were large enough to make a strong correlation between diseases in their gene distribution pattern. Indeed, among these 6 cell types, microglia showed the most similar changes in gene expression among these three neurodegenerative diseases.

Astrocyte and endothelia genes also showed significant positive correlations, suggesting similar disease-related gene expression changes in these cell types between MS and PD (Fig 3). On the other hand, this strong correlation in astrocytes and endothelia cells was not observed in MS vs. AD (Fig 2) or in AD vs. PD (Fig 4), mainly because the changes in AD were subtle and not significant (Fig 1). Thus, unlike microglia genes, the similarity in gene expression changes in astrocyte and endothelial cells exists only between MS and PD, while AD differs.

Neuronal and oligodendrocyte gene expression changes showed weak correlations among diseases (Figs 1, 2, 3 and 4). Thus, compared to the other three cell types (microglia, astrocyte, and endothelia), gene expression changes in neurons and oligodendrocytes are more unique among the three diseases.

### Gene expression in shared cell types

Table 1 shows the commonly upregulated genes in all three diseases in microglia, as well as genes that shared the upregulation feature between MS and PD in astrocyte and endothelia cells.

**Table 1:** Microglia, astrocyte, and endothelia genes with most gene expression changes by disease in AD, MS, and PD. The selection threshold of log2 disease/normal ratios was 0.5< (bold).

	Gene	Log_2_(Disease/Normal) ratio			
		AD	MS	PD	
Microglia	*ALOX5AP*	**0.79**	**0.70**	**1.15**	Common in 3 diseases
	*C1QA*	**0.66**	**0.79**	**1.47**	
	*C1QB*	**0.75**	**0.81**	**1.20**	
	*C1QC*	**0.58**	**0.79**	**1.17**	
	*CD74*	**0.66**	**0.59**	**0.85**	
	*FCER1G*	**0.65**	**0.84**	**0.92**	
	*HSPA1A*	**0.63**	**1.19**	**1.04**	
	*ITGB2*	**0.64**	**0.69**	**0.87**	
	*SPP1*	**0.73**	**1.06**	**1.62**	
Astrocyte	*DTNA*	0.13	**0.92**	**0.56**	Common in MS and PD
	*GJA1*	0.24	**0.68**	**0.73**	
	*ID4*	0.20	**0.56**	**0.60**	
	*KCNN3*	-0.02	**0.57**	**0.70**	
	*MGST1*	-0.02	**0.88**	**0.51**	
	*MUC1*	0.01	**1.13**	**0.51**	
	*PALLD*	0.14	**0.59**	**0.95**	
	*PAX6*	0.14	**0.54**	**0.59**	
	*PDK4*	0.30	**0.83**	**1.13**	
	*SLC1A3*	0.00	**0.52**	**0.83**	
	*SLC7A2*	0.33	**0.81**	**0.54**	
	*SOX9*	0.19	**0.69**	**0.82**	
Endothelia	*AHNAK*	0.24	**0.59**	**0.85**	Common in MS and PD
	*ANKRD37*	0.10	**0.50**	**0.82**	
	*C10orf10*	0.21	**0.81**	**0.71**	
	*HSPB1*	0.44	**0.86**	**1.11**	
	*IFITM2*	0.08	**0.89**	**0.91**	
	*IFITM3*	0.11	**0.90**	**1.14**	
	*LRRC32*	0.11	**0.73**	**0.50**	
	*PECAM1*	0.16	**0.57**	**0.98**	
	*S100A11*	0.32	**0.59**	**0.90**	
	*TGFBR3*	0.23	**0.55**	**0.69**	
	*TIMP1*	-0.09	**0.90**	**0.77**	
	*TM4SF1*	0.14	**0.78**	**1.12**	
	*TPD52L1*	0.29	**0.63**	**1.14**	

## Discussion

Using human superior frontal gyrus microarray data from multiple sclerosis, Parkinson’s disease, and Alzheimer’s disease, we performed a comparative analysis to determine the relationship between these three diseases. Overall, we report two approaches to determine the relationship of gene expression changes among MS, AD, and PD: First, comparison of gene expression changes using boxplot (Fig 1), and second, correlation coefficient tests of changes between two diseases (Figs 2, 3 and 4). The comparison of gene expression changes in MS, PD, and AD showed similarities and differences between these three distinct neurodegenerative diseases in a cell type specific manner. These results cross-validate each other and lead to several conclusions. First of all, microglia genes are quite similar among MS, AD, and PD in terms of disease-related gene expression changes. It is likely that many of the same genes are upregulated in disease in this cell type. Secondly, disease-related behaviors of astrocytes and endothelial cell genes are similar between MS and PD, but not between MS and AD. Also, the influence of disease in neurons shows down-regulation in all three diseases, however changes in the individual neuronal genes are only partially shared between diseases.

Among CNS cell types examined here, microglia showed the most similarity between MS, AD, and PD. Recently, Yokoyama et al [18] reported that several genetic variants leading to increased disease risk are common between AD and immune mediated diseases. In addition, Skene and Grant [19] showed that AD and MS associated genes are commonly enriched with microglial genes, consistent with our observation of common upregulation of microglial genes in MS and AD. Thus, these two distinct diseases could be rooted in similar fundamental molecular mechanisms in microglia. Whether activated microglia are primary or reactive to neuronal and synaptic damage in AD and MS remains unknown, with the two possibilities not mutually exclusive. However, Chanaday and Roth [20] reported the activation of microglia in rat frontal cortex during the first hours of EAE onset, in the absence of detectable peripheral immune cell infiltration, suggesting an initiative role of activated microglia in MS. Also, using an AD mouse model, potential involvement of microglia in early synaptic loss before plaque formation was reported [21]. These observations suggest a causative role. However, causality cannot be shown by our findings herein, since it is nearly impossible to show causality in human studies unless an intervention such as a clinical trial is conducted. That said, genes with upregulated expression in microglia could represent candidate targets for treatment in the early stages of these neurodegenerative diseases. However, beneficial effects of microglia must also be considered [22], so a clinical trial aiming to decrease microglial activation would need to not only test for potential beneficial effects of an intervention, but also monitor closely for potential deleterious effects which may occur depending on timing and other factors during disease [23].

Upregulation of complement protein genes (*C1QA*, *C1QB*, and *C1QC*) was common in the microglia gene group in all three diseases. While these proteins are involved in synaptic pruning during normal brain development, neurodegenerative diseases also show upregulation of these genes [24–31], suggesting that upregulated expression of complement proteins could contribute to the neuropathogenesis of these diseases in adults [32]. A deleterious role of complement activation in AD has been suggested [33, 34]. In an AD mouse model, C1q was upregulated in microglia and mediated the early synaptic loss [21]. In their study, oligomeric β-amyloid mediated synaptic loss was not induced in C1qa knockout mice or in wild-type mice treated with anti-C1q antibody. Also, in a glaucoma mouse model, synaptic loss was absent in *C1qa* knock out mice, and treatment with a complement component 1 inhibitor reduced eye pathology [35]. In addition, in viral encephalitis mediated memory impairment, C1qa was upregulated, and mice deficient in either C3 or C3a receptor had reduced synaptic terminal loss [36]. Thus, the complement activation pathway contains attractive pharmacological targets, and our data suggest that this could be applied to MS, PD, and AD.

Another gene, *ITGB2* (*Integrin Subunit Beta 2*), previously known as *CD18*, is expressed in microglia, with increased expression in AD brain compared to normals [37]. *ITGB2* is the β-chain component of heterodimeric receptors (β2-integrins), and together with α-chains (CD11a-d), β2-integrins are involved in ligand binding and signaling events [38]. In MS, these β2-integrins have been considered as therapeutic targets [39, 40], however, this therapeutic approach was complicated by undesirable side effects [38]. Nonetheless, similar upregulation patterns of this gene in MS, PD, and AD validate our approach and provide proof of principle for strategies designed to therapeutically alter expression of this gene in both diseases if off target effects can be minimized.

A limitation of our study is that we used gene expression data of superior frontal gyrus for the three diseases. Thus, cell type specific similarities and differences here could vary in other brain regions. In AD, hippocampus is the brain region where damage begins, while the substantia nigra is the key region in PD. Damage in MS is postulated to start in multiple white matter focal areas of the brain and spinal cord. Thus, in all three diseases, superior frontal gyrus is a secondary brain region affected by initial damage elsewhere. Despite the fact that the superior frontal gyrus is not initially affected in these diseases, it often eventually becomes involved. Whether microglia play a causal role in the evolution of abnormalities across the brain as these diseases progress remains unknown. If this were true, then halting the spread of neuropathology across brain regions by microglia could represent a therapeutic opportunity.

In summary, similarities and differences in disease-related gene expression in the brain are cell type specific. Among six CNS cell types, microglia shows the highest similarity among these neurodegenerative diseases. Repurposing of drugs for a disease that has FDA approval for another disease represents a rapid and relatively inexpensive pathway to new drug discovery. Our data indicate that cell specificity in similarity of gene expression may be a critical determinant of which treatments may be repurposed. Treatments targeting shared gene expression changes in microglia are warranted in MS, PD, and AD, while treatments targeting astrocyte and endothelia could be shared in MS and PD. On the other hand, those that target the other cell types would likely need to be tailored specifically to each disease.

## Supporting information

S1 Table CNS cell type enriched genes and gene expression changes by disease. (XLSX)
